# High-Throughput Computing Assisted by Knowledge Graph to Study the Correlation between Microstructure and Mechanical Properties of 6XXX Aluminum Alloy

**DOI:** 10.3390/ma15155296

**Published:** 2022-08-01

**Authors:** Xiaoyu Zheng, Yi Kong, Tingting Chang, Xin Liao, Yiwu Ma, Yong Du

**Affiliations:** 1 State Key Laboratory of Powder Metallurgy, Central South University, Changsha 410083, China; xiaoyu_zheng@csu.edu.cn (X.Z.); yong-du@csu.edu.cn (Y.D.); 2 College of Computer Science and Electronic Engineering, Hunan University, Changsha 410082, China; tingtingchang@hnu.edu.cn (T.C.); xinliao@hnu.edu.cn (X.L.); 3 National Supercomputing Center in Changsha, Hunan University, Changsha 410082, China; myw@hnu.edu.cn

**Keywords:** knowledge graph, high-throughput computing, microstructure design, crystal plasticity, Al alloys

## Abstract

It is of great academic and engineering application to study the evolution of microstructure and properties of age-strengthened aluminum alloys during heat treatment and to establish quantitative prediction models that can be applied to industrial production. The main factors affecting the peak aging state strength of age-strengthened aluminum alloys are the precipitates, solid solution elements, grain size effects, and textures formed during the material processing. In this work, these multi-scale factors are integrated into the framework of the knowledge graph to assist the following crystal plasticity finite elements simulations. The constructed knowledge graph is divided into two parts: static data and dynamic data. Static data contains the basic properties of the material and the most basic property parameters. Dynamic data is designed to improve awareness of static data. High-throughput computing is performed to further obtain clear microstructure-property relationships by varying the parameters of materials properties and the characteristics of the structure models. The constructed knowledge graph can be used to guide material design for 6XXX Al-Mg-Si based alloys. The past experimental values are used to calibrate the phenomenological parameters and test the reliability of the analysis process.

## 1. Introduction

Aluminum alloy combines the advantages of low density, good electrical conductivity, high corrosion resistance, good heat dissipation, high specific strength, and easy processing and is widely used in transportation, aerospace, and other industries [1,2,3]. The relationship between “process-structure-performance” of aluminum alloy industrial production is quite complex. Specifically, the process includes determining the composition, heat treatment, deformation processing, etc.; the structure involves grain shape and orientation, composition segregation, and second equivalence. The performance includes elasticity, plasticity, fracture toughness, etc.

The concept of Integrated Computational Materials Engineering (ICME) [4] was introduced by the US government in 2008 to integrate the tools of computational materials science into a holistic and systematic materials development process to achieve efficient development, manufacturing, and use of advanced materials by bridging the gap between materials design and manufacturing. ICME is now widely recognized and adopted by industry and academia and will play a significant role in materials development.

The primary strengthening mechanism of 6xxx-series aluminum alloys is the obstruction of dislocation movement by second-phase particles precipitated during aging, and microstructural parameters such as morphology, size, number, and distribution of the second phase determine the strengthening effect [5,6]. The precipitation process of the second phase is mainly influenced by the process parameters such as alloy composition, aging temperature, and aging time. By establishing a quantitative model between process parameters and microstructure and correlating microstructure parameters with alloy properties, the influence of process parameters on alloy properties can be quantitatively studied, which is of great value for the rational design of alloy composition, optimization of heat treatment conditions, and improvement of alloy properties.

**Figure 1:**
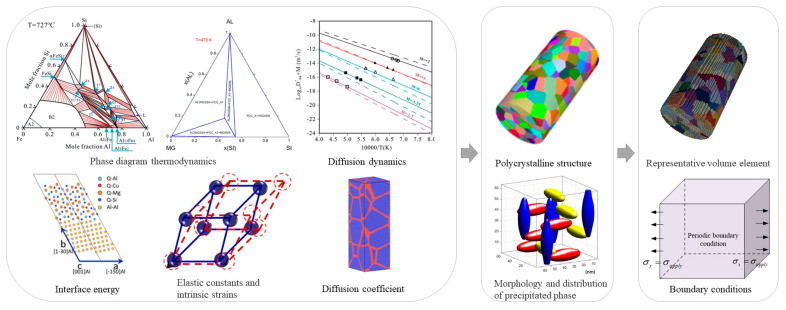
Schematic diagram of multi-scale calculations.

In general, the macroscopic mechanical properties of a material depend on the microstructure, spanning several scales from micro to macro. A complete multi-scale simulation starts from first-principles calculations, molecular dynamics, and Monte Carlo simulations to calculate material physical property parameters such as elastic constants, intrinsic strains, interfacial energies, diffusion coefficients, etc. Then around the specific production process parameters, based on the phase diagram thermodynamics and kinetics to summarize the phase transition law, using the phase-field method, the meta-cellular automata method can be obtained microstructure. Finally, the stress-strain behavior of the material is simulated using finite elements. Figure 1 shows a schematic of multi-scale calculations, from component design through performance simulation, and finally improving component design based on performance simulation results.

## 2. Knowledge Graph

**Figure 2:**
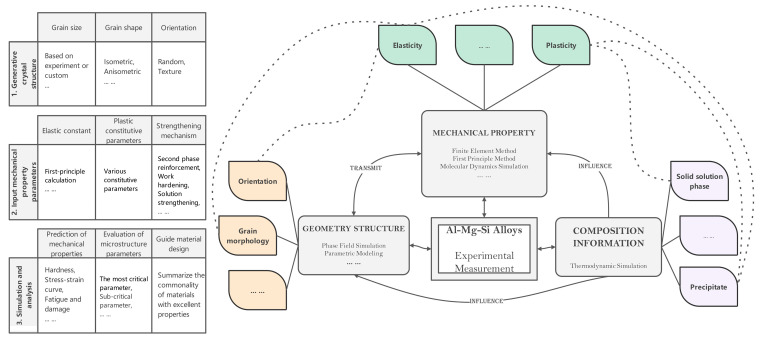
Static data, which calculations can be performed for a specific material, what method is used for each analysis, and the selection of material parameters used for the calculation.

**Figure 3:**
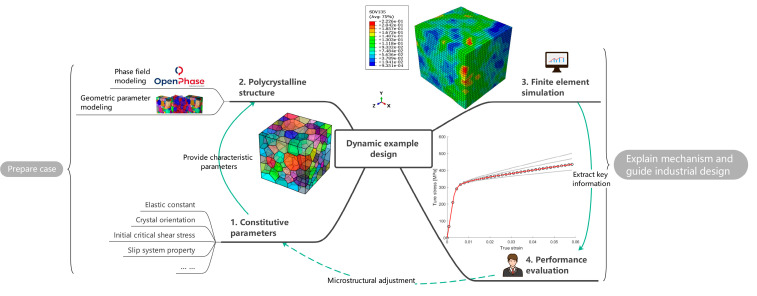
Schematic diagram of dynamic data, indicating specific simulation experiments based on static data of specific materials, what types of calculations are performed in the simulation experiments, and the relevant software and steps required for specific calculations.

The knowledge graph is a semantic network composed of nodes and edges that map the real world to the data world. Nodes represent entities or concepts in the physical world, and edges represent entities’ attributes or relationships [7]. A knowledge graph was first proposed by Google in 2012 [8] for better serving searches. Nowadays, the application fields of knowledge graphs are becoming wider and wider. In the face of finance, medical and other industries, it is also possible to construct knowledge graphs belonging to specific fields. Through information acquisition, knowledge fusion, and knowledge processing, the facts in the original data are refined, analyzed, and formed into a graph. The machine can find the potential associations in the complex relationship and complete the work of case analysis and anti-fraud. In traditional material calculation, experimental data is scattered. The calculation data of a single process is stored and analyzed separately. We calculate the correlation between data by analyzing materials, obtaining material-oriented related laws and knowledge, and establishing a material knowledge graph. The specific implementation process is as follows: mining the input factors and result in performance/property sets and their corresponding relationships of each link in the material simulation calculation process, constructing the corresponding knowledge graph structure according to the association relationship, and filling the experimental calculation data into the knowledge graph structure through mining analysis and processing to form the knowledge graph. Based on the material knowledge graph, reasonable input parameter value recommendations can be provided for actual simulation calculations. Based on the analysis process shown in Figure 1, we divide the aluminum alloy simulation calculation process data into two categories: one is static data such as key performance, computational simulation methods, and basic simulation elements and simulation steps (Figure 2). The other is the input and output data of the software that can be changed in the processing flow, which we call dynamic data (Figure 3). The static data part mainly reflects the relationship between materials and software and calculation types. The active data part mainly records the calculation process data.

For 6XXX series alloys, the components in the static data are extracted based on the concerned work hardening to form a complete calculation example process. Starting from the selection of material parameters, the geometric model is constructed, the calculation example is prepared, the simulation results are obtained, and the results and parameters are analyzed. Finally, the parameters are evaluated and a solution for material performance improvement is obtained. The calculation results of dynamic data will be used to increase or decrease static data, such as by introducing new methods and strategies, considering more microstructure information, or discarding unimportant microstructure information.

## 3. Material Modeling

In this section, the basic framework of micromechanical modeling is introduced in detail. The described model consists of a geometric description of the grain structure of polycrystals. The present construction model of plastic deformation in a single grain is realized by the crystal plasticity method through the user-defined material model (UMAT) of ABAQUS.

### 3.1. Representative Volume Elements

When simulating the properties of a material, the results are undoubtedly most accurate if the model constructed covers all the information about the material but requires a large number of calculations beyond the current level of computer development. The representative volume element (RVE) is a unit that is much smaller than the macroscopic system of the material but is large enough to capture the basic characteristics of the microstructure. The construction of representative volume cells with various structural features enables rapid analysis of the effects of material microstructural changes on performance.

There are two main strategies to construct representative volume elements, one is to use experimental characterization techniques such as electron backscatter diffraction (EBSD) to obtain real microstructures and thus build geometric models, and the other is to get microstructures using phase-field simulations, Monte Carlo methods, Voronoi, etc.

**Figure 4:**
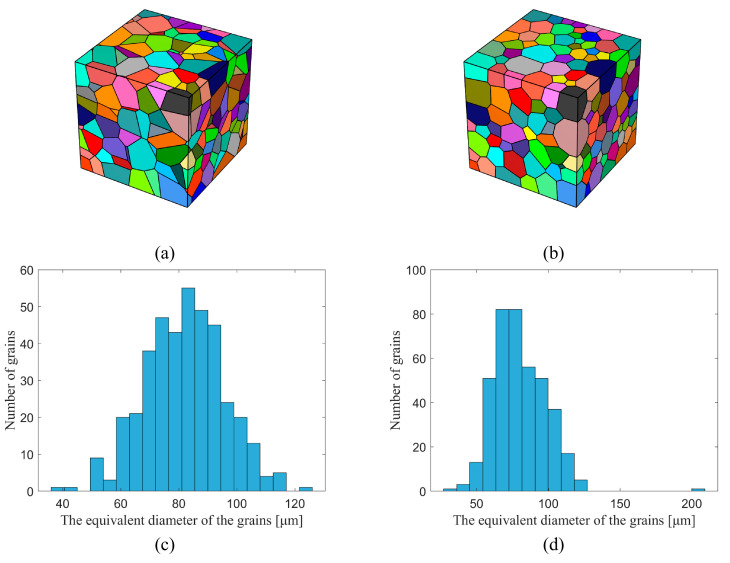
RVE of two different grain shapes, both cubes with 500 µm side length and containing 399 grains. (**a**) The shape of the grains is Voronoi polyhedral. (**b**) The shape of the grains is polyhedral with high sphericity. (**c**,**d**) are the equivalent diameter distributions of grains in RVE in (**a**,**b**), respectively.

The first strategy is mainly used to reveal the relationship between microstructure and properties of specific materials. Depending on the research problem, it can be either by modeling the high matching of the observed region or by extracting statistical information from the EBSD observations to construct RVE. Based on the EBSD observations, we can obtain information about the grain shape, size, orientation, etc. Luo et al. [9,10] targeted the initial stages of fatigue cracking by constructing RVE directly based on microstructure scans of material samples in regions of high-stress concentration. This approach, which corresponds the RVE exactly to the modeled area, is inherently deterministic but requires a large amount of experimental data and complex preparation, as well as a significant computational effort, and is therefore only used when exploring specific micromechanical mechanisms. When studying physical quantities of macroscopic statistical significance, such as yield stress, tensile strength, and elongation at break at the visible level, information such as grain features, including orientation, disorientation, and grain size, can be extracted from microstructural observations to construct RVE. This statistical information-based modeling approach can be easily implemented by many software programs, such as Neper [11,12,13], DREAM.3D [14], and Kanapy [15]. Figure 4 shows two RVE with equiaxed crystal organization constructed by Neper based on the same seed point. Figure 4a shows the standard Voronoi polyhedral structure, and Figure 4b shows the stable system with higher grain sphericity. Figure 4c,d reflects the difference in the size distribution of grain morphology, with a more concentrated grain size distribution in the Voronoi polyhedral structure.

The second strategy is mainly used to find the correspondence between process parameters and properties of the material. The microstructures obtained by simulation-based means are more energy-efficient and faster than preparing alloy samples, and the uncontrollable factors are significantly reduced. Borukhovich et al. [16] combined the phase field approach with crystal plasticity theory to simulate the entire machining cycle from quenching, and over-tempering, to mechanical testing.

**Figure 5:**
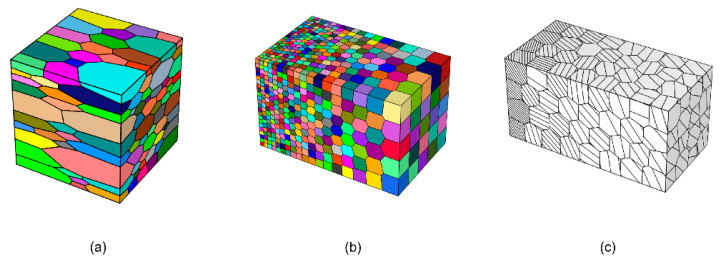
(**a**) Polycrystalline geometry model of nonequiaxed crystal. (**b**) Polycrystalline geometric model with gradient distribution of crystal size. (**c**) Polycrystalline geometric model with a gradient distribution of the number of precipitates.

In addition to constructing representative volume cells that match the real microstructure, it is also possible to explore the influence of material microstructure on properties by constructing representative volume cells with different characteristics, such as grains of specific morphology, the spatial distribution of grain size, and chemical composition, etc. Figure 5a shows the mechanical properties of non-isometric crystals by constructing grains with different aspect ratios, which correspond to aluminum alloys with elongated grains after rolling or unidirectional stretching. Figure 5b,c shows the non-uniform distribution of grains and precipitates composition, respectively.

In reality, aluminum alloys may not always exhibit such distinct gradient structures, but setting these structural variations to be obvious in the simulation allows for a more intuitive exploration of the effects of such structures.

**Figure 6:**
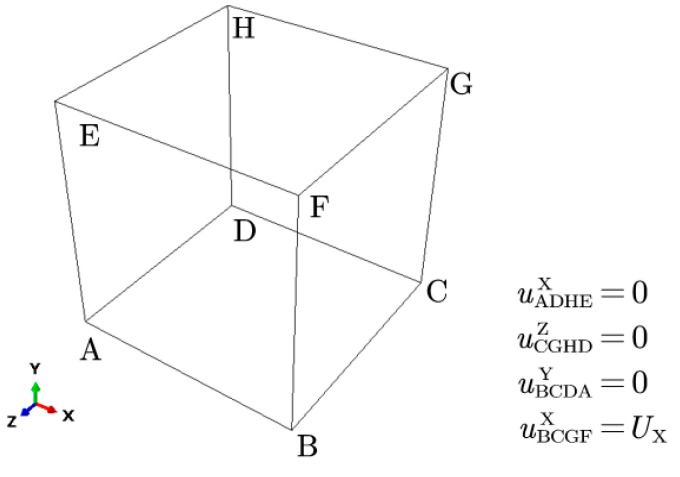
Boundary conditions of uniaxial tension.

By setting the corresponding boundary conditions for representative volume cells, the deformation processes of different materials, such as tension, shear, etc., can be simulated. The purpose of this work is to study the yielding and work-hardening behavior of the material by simulating the uniaxial stretching of the material. In total, the boundary conditions are set on four faces of the RVE, as shown in Figure 6.

### 3.2. Crystal Plasticity Model

The material behavior simulated by the finite element method is described by a phenomenological crystal plasticity model. In order to address the non-uniform deformation caused by abrupt changes in the mechanical behavior of polycrystalline grain boundaries and to consider the effect of crystallographic textures, the single crystal constitutive model proposed by Asaro [17] is used. In this paper, vectors (lowercase letters) and second-order tensor matrices (uppercase letters) are indicated in bold.

The deformation kinematics theory points out that the total deformation gradient F can be decomposed by multiplication and expressed as a combination of $F^{e}$ and $F^{p}$: $F=F^{e}F^{p}$. The elastic deformation of the material follows Hooke’s law. Plastic deformation is mainly calculated by the plastic strain gradient $l^{p}$, which is a function of the plastic deformation gradient $F^{p}$:

(1) $$l^{p}={\dot{F}}^{p}F^{p-1}$$

Assuming that slip is the only displacement mechanism of plastic deformation, $l^{p}$ can be expressed as the sum of the shear rates of all slip systems:

(2) $$l^{p}=\sum_{\alpha }{\dot{\gamma }}^{\alpha }s^{*\alpha }⊗m^{*\alpha },$$

where ${\dot{\gamma }}^{\alpha }$ is the plastic shear rate and $s^{*\alpha }⊗m^{*\alpha }$ is the Schmid tensor of the slip system α, which is obtained by dyadic operation between the slip direction $s^{*\alpha }$ and the normal direction of the slip surface $m^{*\alpha }$. Aluminum is Face-Centered Cubic (FCC) crystal, the value of α is 1 to 12. According to the power law model proposed by Asaro et al. [17], the plastic shear rate of α slip system can be expressed as:

(3) $${\dot{\gamma }}^{\alpha }={\dot{\gamma }}_{0}{\left(\frac{\left|\tau^{\alpha }\right|}{g^{\alpha }}\right)}^{1/m}\mathrm{sgn}\left(\tau^{\alpha }\right),$$

where ${\dot{\gamma }}_{0}$ is the reference shear rate, $g^{\alpha }$ is the plastic deformation resistance, and the resolved shear stress $\tau^{\alpha }$ is the projection of the Kirchhoff stress tensor def(F)σ onto the slip surface. The parameter *m* controls the sensitivity of the strain rate. Assuming that the density of the material remains constant during the deformation process, $\tau^{\alpha }$ can be expressed by the stress tensor σ:

(4) $$\tau^{\alpha }=m^{*\alpha }\cdot \sigma \cdot s^{*\alpha }$$

The initial value of $g^{\alpha }$ is initial slip resistance $\tau_{i}$, which is assumed to be the same for all slip systems. Work hardening is introduced by making the resistance to plastic deformation a function of plastic strain:

(5) $${\dot{g}}^{\alpha }=\overset{12}{\underset{\beta =1}{\sum }}h_{\alpha \beta }\left|{\dot{\gamma }}^{\beta }\right|,$$

where $h_{\alpha \beta }$ is the hardening modulus matrix, $h_{\alpha \alpha }$ denotes the hardening due to the slip of its own slip system, usually called the self-hardening coefficient, and $h_{\alpha \beta }\left(\alpha \neq \beta \right)$ denotes the hardening due to the slip of other slip systems, usually called the latent hardening coefficient. q is the ratio of the latent hardening coefficient to the self-hardening coefficient, 1 and 1.4 are the more common values. The $h_{\alpha \beta }$ can be expressed as a unified equation:

(6) $$h_{\alpha \beta }=h\left(\alpha ,\alpha \right)\left[q+(1-q)\delta_{\alpha \beta }\right]=\{\begin{array}{cc} h\left(\alpha ,\alpha \right) & \beta =\alpha \\ qh\left(\alpha ,\alpha \right) & \beta \neq \alpha \end{array}$$

Self-hardening coefficients using the model adopted by Peirce et al. [18]:

(7) $$h\left(\alpha ,\alpha \right)=h_{0}{\mathrm{sech}}^{2}\left(\frac{h_{0}\gamma }{\tau_{s}-\tau_{i}}\right)=h_{0}{\mathrm{sech}}^{2}\left(k\gamma \right),$$

where $h_{0}$ is the hardening modulus at the beginning of yield, $\tau_{s}$ is the plastic flow breakthrough stress in the first stage of the material, and $\gamma =\sum_{\alpha }\int_{0}^{t}\left|{\dot{\gamma }}^{\alpha }\right|dt$ is the cumulative shear strain of each slip system. Noting that $h_{0}/\left(\tau_{s}-\tau_{i}\right)$ is a constant value, the softening factor *k* can be introduced to visualize the effect of γ on the degree of hardening of the material.

The region of large plastic deformation during material deformation may become the location where cracks sprout, and the location of material failure can be predicted by introducing accumulated plastic deformation p [19]:

(8) $$p=\int_{0}^{T}{\left(\frac{2}{3}l^{p}:l^{p}\right)}^{\frac{1}{2}}dt$$

In addition, the local plastic dissipation energy $E_{p}$ [20,21] can also provide a prediction of material damage:

(9) $$E_{p}=\int_{0}^{T}\sigma :l^{p}dt=\underset{\alpha }{\sum }\int_{0}^{T}\tau^{\alpha }{\dot{\gamma }}^{\alpha }dt$$

From the FE simulation, ABAQUS gives the value of each physical quantity at the center-of-mass for each element, (∙). The black dots in parentheses indicate the homogenized parameters, i.e., stress and strain, etc. To obtain a global representative value for each time step, the center-of-mass values of each element are averaged through the element volume. For example, volume averaging of stresses and strains (${\bar{\sigma }}_{ij}^{\mathrm{RVE}}$ and ${\bar{\epsilon }}_{ij}^{\mathrm{RVE}}$) can be performed for comparing the stress-strain curves obtained from the tests.

(10) $$\begin{array}{c} {\bar{\sigma }}_{ij}^{\mathrm{RVE}}=\frac{1}{V^{\mathrm{RVE}}}\overset{N}{\underset{n=1}{\sum }}{\left(\sigma_{ij}\right)}_{n}\cdot V_{n}\\ {\bar{\epsilon }}_{ij}^{\mathrm{RVE}}=\frac{1}{V^{\mathrm{RVE}}}\overset{N}{\underset{n=1}{\sum }}{\left(\epsilon_{ij}\right)}_{n}\cdot V_{n} \end{array},$$

where the subscript n represents the value of each unit, and $V^{\mathrm{RVE}}$ represents the total volume of the entire RVE. This averaging strategy can be applied equally to p and $E_{p}$ to measure the deformation properties of the material.

In addition, the Lode stress parameter $\mu_{\sigma }$ can be introduced to analyze the stress state of the material during deformation. Notice that $\mu_{\sigma }$ is equal to -1 in uniaxial tension and 0 in pure shear. The Lode stress parameter for an RVE ${\bar{\mu }}_{\sigma }^{\mathrm{RVE}}$ is the volume average of each element $\mu_{\sigma }$:

(11) $$\mu_{\sigma }=\frac{\sigma_{2}-\left(\sigma_{1}+\sigma_{3}\right)/2}{\left(\sigma_{1}-\sigma_{3}\right)/2},$$

where $\sigma_{1}$, $\sigma_{2}$, $\sigma_{3}$ are the three principal stresses ($\sigma_{1}⩾\sigma_{2}⩾\sigma_{3}$), which can be obtained from the stress tensor σ.

### 3.3. Strength Model

In the crystal plasticity model proposed in the previous section, the most important parameter is $\tau_{i}$. There are many strengthening models [5,22,23] for 6XXX series aluminum alloys, which aim to relate microstructural parameters, such as grain size, texture, size distribution of precipitates, type, and content of solid solution phases, to macroscopic yield strength $\sigma_{y}$. Contributions are usually linearly additive [5,6]:

(12) $$\sigma_{y}=\sigma_{\mathrm{Al}}+\sigma_{\mathrm{ss}}+\sigma_{\mathrm{ppt}}$$

The intrinsic strength, solid solution contributions, and precipitates to the yield stress of aluminum are denoted as $\sigma_{\mathrm{Al}}$, $\sigma_{\mathrm{ss}}$ and $\sigma_{\mathrm{ppt}}$, respectively. Similarly, at the single crystal level, the initial slip resistance $\tau_{i}$ is determined by various microstructural parameters:

(13) $$\tau_{i}=\tau_{\mathrm{Al}}+\tau_{\mathrm{ppt}}+\tau_{\mathrm{ss}}$$

$\tau_{\mathrm{Al}}$ stands for intrinsic strength of aluminum, which is numerically equal to 3/1 of the yield strength of pure aluminum with the same average particle size. Therefore, in this model, the grain size effect is also considered.

The solid solution strengthening term $\tau_{\mathrm{ss}}$ is due to the strain field generated around the substitutional atoms dissolved in the matrix that can interact with dislocations and impede their movement, resulting in strengthening. Based on the principle that the contributions of different solute atoms to the yield strength can be linearly superimposed, the solid solution strengthening effect of the alloy can generally be expressed as:

(14) $$\tau_{\mathrm{ss}}=\sum_{i}k_{i}C_{i}^{2/3},$$

where $k_{i}$ is the scaling factor and $C_{i}$ is the mass fraction (wt.%) of the specific element (Mg, Si) in the solid solution. The value of $C_{i}$ is easily known based on the thermodynamics of the phase diagram or on the quantitative chemical analysis of the alloy structure. According to the work of Myhr et al. [24], $k_{\mathrm{Mg}}$ and $k_{\mathrm{Si}}$ take the values of $15.0\mathrm{MPa}/\mathrm{wt}.{\%}^{2/3}$ and $33.0\mathrm{MPa}/\mathrm{wt}.{\%}^{2/3}$, respectively.

Unlike $\tau_{\mathrm{Al}}$ and $\tau_{\mathrm{ss}}$, which have clear and unambiguous expressions, modeling $\tau_{\mathrm{ppt}}$ is always extremely difficult. Esmaeili et al. [22] proposed that the reinforcement of the precipitated phase is related to several microscopic variables, which can be expressed as:

(15) $$\tau_{\mathrm{ppt}}=ℱ\left(r,f,F,l,S\right)$$

where r and f are the average size and volume fraction of the precipitates, respectively, F is the maximum interaction force between the particle and dislocation with the average radius, l is the average distance between the particles of the precipitates with obstruction, and S is a series of microscopic parameters indicating the particle shape of the precipitates and the dislocation relationship between the particle and the matrix. This idea was widely adopted and promoted in the following decades [6,23,25,26]. Because the types, morphology and distribution of the precipitates depend on the processing technology and the composition of the raw materials, it is not easy to construct a clear functional relationship ℱ. Therefore, the $\tau_{\mathrm{ppt}}$ can be considered to be determined preferentially by Equation (13):

(16) $$\tau_{\mathrm{ppt}}=\tau_{i}-\tau_{\mathrm{Al}}-\tau_{\mathrm{ss}}$$

In most studies, the yield stress and the initial slip resistance are considered to satisfy a linear relationship: $\sigma_{y}=M\tau_{i}$, where *M* is the Taylor factor, which is the most important parameter connecting the continuum plasticity theory and the crystal plasticity theory. For specific materials, the measurement of yield stress $\sigma_{y}$ is very convenient, so $\tau_{i}$ can be estimated by Taylor factor *M*:

(17) $$\tau_{i}=\frac{\sigma_{y}}{M}$$

For randomly oriented FCC structured metals, the value of *M* is 2.2 for the Sachs model [27] and 3.1 for the Taylor model [28]. The work of Zhang et al. [29] states that the value of *M* when using the crystal plasticity finite element model is about 2.7. In fact, *M* is closely related to the textures of the material and is an important physical quantity that characterizes the statistical significance of the crystallographic orientation.

### 3.4. Parameter Calibration

Although the above model has, as far as possible, covered all the factors most commonly considered in the study of the strength of 6XXX series aluminum alloys. However, due to the complex process conditions, composition ratios, and the complexity of the real microstructure of the material during production, some of the phenomenological or structure-sensitive parameters in the model need to be calibrated to the results of material-specific mechanical properties tests in order to subsequently predict the mechanical response of the material when its microstructural characteristics are altered or under more complex loading conditions. For parameters that are universal and structurally insensitive, such as elastic constants and power-law hardening parameters, the values in the literature are directly selected.

The 6XXX series aluminum alloy under under-aged and peak-aged states reported by Yang et al. [25] was selected for finite element simulation and some key parameters were compared accurately. The purpose of this work was chosen because the two materials reported in the paper only have different aging times, the remaining microstructure parameters are almost identical to the initial alloy composition, they have similar compositions in the knowledge graph, and differences in their mechanical properties are also easy to test whether they can be reflected by specific parameters. Each material is tested for uniaxial tensile, and multiple sets of test values are selected to average to ensure the effectiveness of the test. The specimen used for mechanical testing is cut from a randomly selected location in the casting sample, and the entire specimen is not significantly mechanically processed, so it can be considered that the orientation of the grains is randomly distributed, without texture. Considering that the average grain sizes of the two samples are the same, the same geometric model is constructed as well as the random grain orientation. The RVE is divided into a total of 39304 elements, 34 elements in each direction. The amount of stretch along the X direction is 6% of the side length.

**Figure 7:**
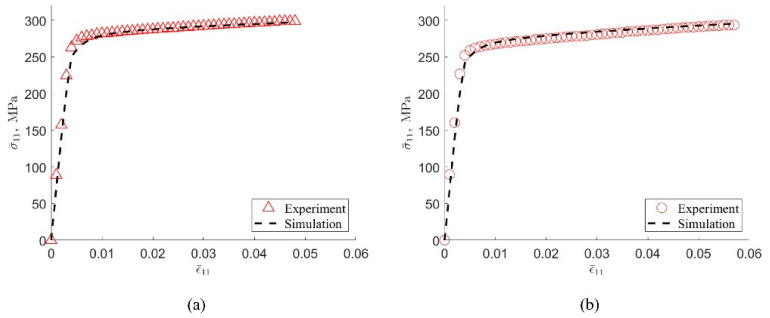
Experimental and simulation results of peak-aged (**a**) and under-aged (**b**) 6XXX series aluminum alloy stress-strain curves.

In this study, the following parameters are based on the literature: $\left(C_{11},\;C_{12},\;C_{44}\left(\mathrm{MPa}\right)\right)=\left(106430,\;60350,\;28210\right)$, $\left[{\dot{\gamma }}_{0}\left(s^{-1}\right),\;m\right]=\left[0.001,\;0.02\right]$. This information exists in the knowledge graph and is used during finite element simulations. Parameters such as $\left[M,\;h_{0},\;k,\;q\right]$ are the key variables that reflect the differences in the properties of each alloy, reflecting the characteristics of the material organization and composition, and fundamentally correspond to different production and processing processes. In addition, $\tau_{i}$ is determined by Equation (17), where the value is taken from the work of Yang et al. [25], as 273.3 MPa for peak-aged alloy and 258.6 MPa for under-aged alloy. M and q are highly correlated with the crystal structure properties and have well-defined intervals, while $h_{0}$ and k are closely related to the precipitation phase properties in the alloy and tend to vary over a wide range, but not by more than one order of magnitude. Based on the trial-and-error method, the approximate range of the values of these parameters is obtained, and then the accurate values are determined by combining the neural network and the genetic algorithm. Figure 7 shows that the stress-strain curve obtained by the finite element simulation is basically consistent with the experiment based on the parameters calibrated by the experiment. Finally, the crystal plastic finite element simulation parameters calibrated based on experiments are summarized in Table 1.

## 4. High-Throughput Computing

The work in the previous chapter has shown that the mechanical behavior of materials can be accurately predicted based on microstructural modeling and parameter calibration. The materials under study can be quantified and recorded in the knowledge graph through physical quantities such as microstructural parameters and mechanical performance parameters. When more experimental data are considered in the future, the knowledge graph will be updated. The background will also continue to become clearer. Another important task of a knowledge graph is to expand new material information and cognition from existing material knowledge. For example, what effect will the combination of various microstructure information have on the performance of the material itself? Which microstructural information will play a more important role? Based on the goal to be explored, a variety of numerical examples can be constructed to study the law of the influence of various parameters on the material properties.

Based on the idea of high-throughput computing, we designed a series of examples to obtain many computational results by varying the values of some parameters of materials properties and considering different initial polycrystal structures for simulation, and summarize the influence weights of each parameter to build a comprehensive mechanical properties-microstructure knowledge graph. The same RVE, Figure 4a, is used for all the calculations. With the material properties either calculated by multi-scale calculations, or using existing material data, high-throughput computing is further performed to achieve efficient screening of composition/organization/performance, etc., to guide the process optimization issues and to accelerate the development of new materials and significantly reduce the cost of material development.

Uniaxial tensile simulations were performed for the model shown in Figure 4a by assigning the same random grain orientation and different material parameters. Table 2 shows the range of values for each parameter, and when a parameter is varied, the default values are set for the remaining parameters. In addition, the effect of crystal orientation on mechanical properties is analyzed by setting different initial textures with constant material parameters taking default values. Table 3 shows the typical common textures of FCC structured metals.

**Figure 8:**
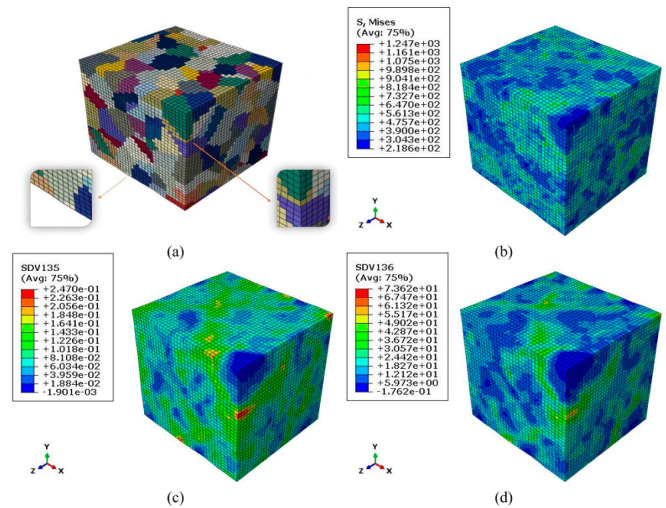
Tensile simulation results for polycrystalline aggregates. (**a**) Grains deformation diagram. (**b**) Distributions of Von Mises equivalent stress. (**c**) Distributions of accumulated plastic deformation. (**d**) Distributions of local plastic dissipation energy.

Figure 8 shows the tensile simulation results for polycrystalline aggregates with material parameters $\left[M,\;h_{0},\;k,\;q\right]$ set to the default values in Table 2. From the Mises stress distribution shown in Figure 8b and the cumulative plastic strain distribution shown in Figure 8c, it can be seen that there are significant differences in stresses and strains among grains, and the stress concentrations are mainly found at grain boundaries. The differences in plastic deformation and stress response between grains are mainly caused by differences in grain orientation, while the differences within grains are mainly caused by grain arrangement and their interactions. The highly anisotropic elastic and plastic behavior of single grains allows the deformation or stress concentration at grain boundaries to satisfy both stress equilibrium and strain compatibility. Therefore, both stress and strain tend to occur at grain boundaries.

**Figure 9:**
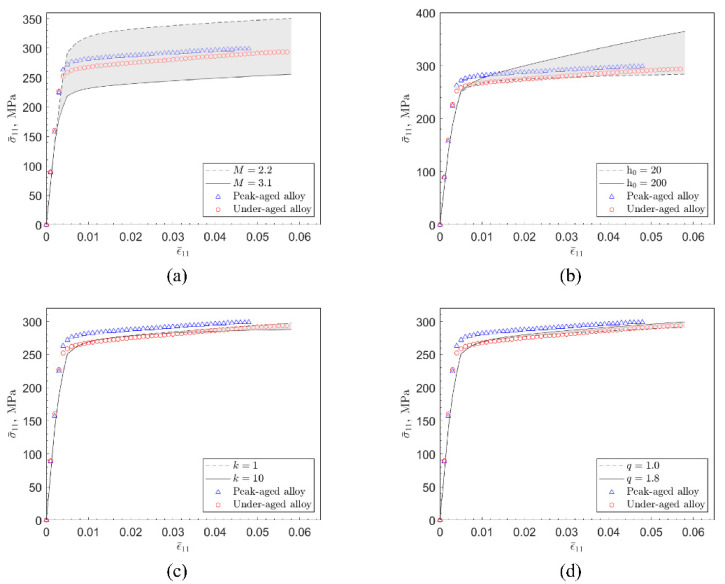
Stress-strain curves of polycrystalline aggregates with different intrinsic parameters ($\left[M,\;h_{0},\;k,\;q\right]$). (**a**) The stress-strain response of the material when M varies from 2.2 to 3.1. (**b**) The stress-strain response of the material when $h_{0}$ varies from 20 MPa to 200 MPa. (**c**) The stress-strain response of the material when k varies from 1 to 10. (**d**) The stress-strain response of the material when q varies from 1.0 to 1.8.

Figure 9 illustrates the calculated results of the stress-strain curve for a single parameter varying according to the values taken in Table 2. The calculated results illustrate that the boundary values of the parameters correspond to the extreme cases of the stress-strain curve, and the gray area in the figure indicates the position of the stress-strain curve of the material when the parameters take intermediate values. M affects the yield stress of the simulation results, $h_{0}$ reflects the strain hardening capacity of the material, k corresponds to the degree of strain hardening of the material, and the variation of q does not bring much difference in the results, thus the correlation between this parameter and the performance is less sensitive. By comparing the experimental values with the calculated results after parameter adjustment, the influence characteristics of these independent parameters on the mechanical properties can be more deeply understood, and the corresponding relationship between them and the knowledge of materials science can be interpreted. M reflects the anisotropy of the material and reflects the orientation correlation between the strength of single crystal and polycrystalline strength. It is more effective to calibrate the initial slip system yield strength based on M than other parameters. When the initial hardening modulus varies between orders of magnitude, the stress-strain response has limited changes in the initial plastic deformation, and then shows a significant difference in work hardening properties. Therefore, this parameter is effective for the characterization of material work hardening, and the difficulty of deformation at the initial stage of material work hardening will significantly correspond to the change in the value of this parameter. After the other parameters are calibrated, the two parameters q and k do not significantly affect the stress-strain response of the material within the common value range, so they may not be suitable for establishing the relationship between microstructure and performance.

**Figure 10:**
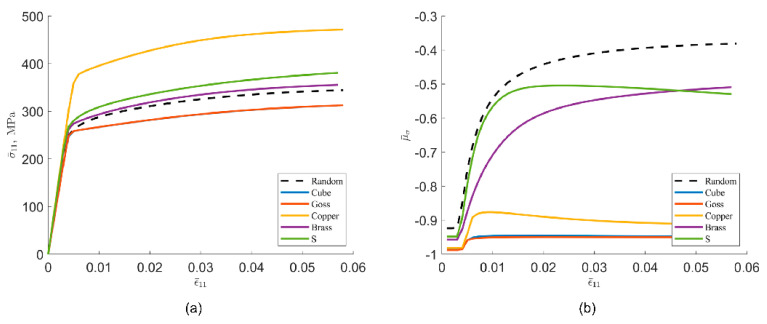
Stress-strain curves of polycrystalline aggregates with different textures ($\left[M,\;h_{0},\;k,\;q\right]$ uses the default values in Table 1). (**a**) Stress-strain curves. (**b**) Lode stress parameter-strain curves.

Figure 10a shows the results of stress-strain simulations for polycrystalline aggregates with different initial crystal orientations, where uniaxial stretching corresponds to the 〈100〉 orientation of the material and thus the ease of grain slip initiation differs for different orientations. This result is consistent with the study of Zhao et al. [30]. Figure 10b shows the effect of the initial texture on the Lode stress parameter-strain curve. The results show that the different oriented materials exhibit significant differences in mechanical properties during deformation in the specified directions.

The rich calculation results obtained by high-throughput calculation provide rich materials for the construction of an aluminum alloy knowledge graph. Based on the objective fact that the structure determines the performance, we set different structures and different material parameters to conduct finite element simulations, and the mechanical responses of various structures can be obtained. The simulation results are stored in the knowledge graph. In the subsequent material development, the key influencing parameters can be located according to the expected mechanical properties, and then key process schemes or material ratios can be found to achieve the accurate material design.

**Figure 11:**
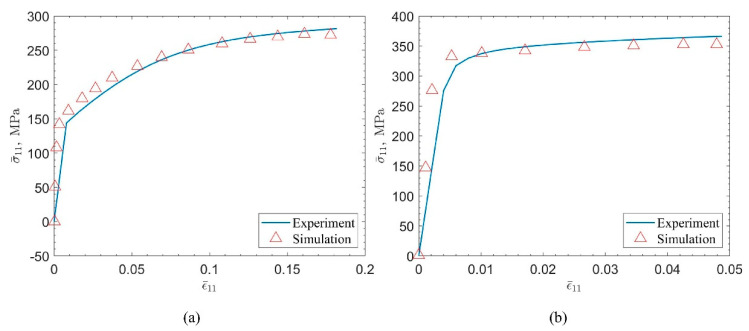
Experimental and simulation results of the stress-strain curve of 6061 aluminum alloy. (**a**) 6061-T4. (**b**) 6061-T6.

Finally, the widely used commercial alloys 6061-T4 and 6061-T6 in the 6XXX series were selected as the research objects, and the materials were selected from the alloy samples with a mass ratio of Mg to Si of 1.19 in the work of Kim et al. [31]. 6061-T4 is an alloy that is naturally aged after solution heat treatment. Its yield stress is approximately 122.0 MPa. This alloy is of average strength but has excellent machinability. The material fails under 20% tensile strain. 6061-T6 is an artificially aged alloy after solution heat treatment. The yield stress is about 325.0 MPa, but the processing performance is poor, and the tensile strain is about 5%. Since the two are obtained from the same batch of alloy samples processed by different aging processes, and the polycrystalline morphology and orientation of the material are basically unchanged during the aging process, it is still assumed that M is the same value, and based on the previous analysis, k and q are also similar. It is considered that it is not sensitive to the structure, and the same value is approximately taken. The final simulation result is shown in Figure 11. The simulation parameters determined based on semi-analytical and semi-experience are as follows. In order to reflect the material analysis strategy based on high-throughput calculation and knowledge graph. Therefore, methods such as machine learning are not used, and the simulation results and the parameters used to reflect the applicability of the previously concluded laws. It also reflects that the initial yield strength $h_{0}$ is a key performance index for 6XXX. The crystal plastic constitutive parameters of the two alloys are listed in Table 4, from which it can be seen that the $\tau_{s}$ of the 6061-T4 alloy is about twice that of the $\tau_{i}$, indicating that the alloy after natural aging has better work hardening ability. If the material has an excellent degree of work hardening at the initial stage of plastic strain, the subsequent deformation performance will also be guaranteed.

## 5. Conclusions

To comprehensively improve the performance of materials and deepen the knowledge of materials undoubtedly requires the ability to simultaneously combine multiple time scales and space scales in simulation calculations, but there is currently no universal method that can cover all time and space scales. Analyzing material properties by synthesizing various factors without screening will result in a huge amount of computation. Therefore, building a knowledge network of various microstructure information-performance of materials based on knowledge graphs will be a major mainstream analysis method in the future.

Integrated computational materials engineering and high-throughput computing will change the traditional empirical trial-and-error approach to alloy research and development (R&D) and become a fundamental R&D platform for collaborative knowledge innovation with the interconnection of multi-scale calculations, experiments, and databases. On this platform, as a three-dimensional data network capable of regularly linking information about the microstructure, properties, and computational methods of materials, the knowledge graph can be used to recommend reasonable input parameter values and store results, thus assisting high-throughput computation. To sum up specifically, for a certain research object, we first summarize and sort out important structural components or key performance control parameters based on previous materials science cognition and experiment-based summary, build a knowledge graph based on the performance of interest, and design a set of analysis and simulation process including these parameters. Multiple sets of examples were designed to study the influence weight of each variable on the performance. Comparing the calculated results with existing experimental observations can explore the relationship between structure and performance on a deeper level, thus reducing the consideration of secondary factors and optimizing the R&D strategy.

6XXX series aluminum alloys are chosen to demonstrate the proposed strategy of studying the correlation between microstructure and mechanical properties by high-throughput computing assisted by a knowledge graph. The simulation results show that the orientation distribution and initial hardening modulus of the material are the main factors affecting its performance, indicating that the previous assumption of isotropy is not suitable for 6XXX series, and how to improve the hardening ability at the initial stage of plastic strain is the focus of research. The crystal plasticity finite element method, as a bridge linking the micro-to-macro to quantitatively describe the relationship between the microstructure and properties of the alloy, is chosen to perform high-throughput computing with varying the parameters of materials properties and the characteristics of the structure models. The constructed knowledge graph is divided into two parts: static data and dynamic data, and can be used to guide material design for 6XXX Al-Mg-Si based alloys. Static data contains the basic characteristics and the most essential characteristic parameters of materials. The purpose of continuous generation and adjustment of dynamic data is to improve the cognition of static data. This research method has universality and popularization value.

## Figures and Tables

**Table 1:** Calibration of crystal plastic finite element simulation parameters based on experimental data.

**Materials**	$C_{11},C_{12},C_{44}\left(\mathrm{MPa}\right)$	$h_{0}\left(\mathrm{MPa}\right)$	$\tau_{i}\left(\mathrm{MPa}\right)$	$\tau_{s}\left(\mathrm{MPa}\right)$	${\dot{\gamma }}_{0}\left(s^{-1}\right)$	m	M	k	q
Peak-aged	106430, 60350, 28210	27.54	101.81	110.99	0.001	0.02	2.7	3.0	1.4
Under-aged	106430, 60350, 28210	44.25	97.75	110.39	0.001	0.02	2.7	3.5	1.4

**Table 2:** Range of variation of material parameters.

Parameter	Default Value	Minimum	Maximum
M	2.7	2.2	3.1
$h_{0}\left(\mathrm{MPa}\right)$	44.25	20	200
q	1.4	1.0	1.8
k	3.5	1	10

**Table 3:** Euler angles of typical textures in FCC metals.

**Type**	{hkl}	uvw	$\varphi_{1}$	ϕ	$\varphi_{2}$
Cube	001	100	0°	0°	0°
Goss	011	100	0°	45°	0°
Copper	112	111	90°	35°	45°
Brass	011	211	35°	45°	0°
S	123	634	59°	37°	63°

**Table 4:** 6061 aluminum alloy crystal plasticity finite element simulation parameters.

**Materials**	$C_{11},C_{12},C_{44}\left(\mathrm{MPa}\right)$	$h_{0}\left(\mathrm{MPa}\right)$	$\tau_{i}\left(\mathrm{MPa}\right)$	$\tau_{s}\left(\mathrm{MPa}\right)$	${\dot{\gamma }}_{0}\left(s^{-1}\right)$	m	M	k	q
6061-T4	106430, 60350, 28210	200.0	47.0	87.0	0.001	0.02	2.6	5.0	1.4
6061-T6	106430, 60350, 28210	50.0	125.0	133.33	0.001	0.02	2.6	6.0	1.4

## Data Availability

The data presented in this study are available on request from the corresponding author.
